# Supplementary material for: Contractile and mechanical properties of hamstring muscles measured by the method of tensiomyography (TMG) in professional soccer players: A systematic review, meta-analysis and meta-regression
Source: PLoS One. 2025 Mar 4;20(3):e0315812. doi: 10.1371/journal.pone.0315812 (PMC11878904; doi:10.1371/journal.pone.0315812)
Supplement: Attachment — Submitted filename: Comments 18.08.2024.pdf [file pone.0315812.s003.pdf]

Comments on:

"Muscle contractile properties of hamstrings in professional soccer players: A systematic review and meta-analysis"

First, I would like to congratulate the authors on selecting a topic that holds significant potential. I believe this study can effectively inform football practitioners about TMG normative values for hamstring muscles, which may impact training optimization. In the submitted article, the authors aimed to: 1) gather scientific evidence on hamstring TMG parameters in professional football players during the season, and 2) establish the most frequent values for the main TMG parameters in soccer players, comparing these with the reference values provided by the TMG software. The results showed slight but significant discrepancies between the reference values from the TMG software and those identified in the literature. While I find this article very interesting for readers, several important issues must be addressed before it can be accepted for publication.

General comments:

- The introduction section lacks a clear problem identification based on previously published literature investigating similar topics. Proper problem identification is crucial, as it provides a solid foundation for why the study is needed. When constructing the narrative, the authors frequently jump from one topic to another, often unrelated to their main aim. By the end of the introduction, it feels as though they abruptly shift from the rest of the introduction to discussing the limitations of current literature—an issue not previously addressed. This is particularly confusing for readers trying to understand the background and the rationale behind comparing TMG reference values provided by the software with those found in published literature.

- In the introduction section, there are several references to the usefulness of TMG as a device that can aid in injury prevention (e.g., Lines 29, 32, 44). While a theoretical framework suggesting that muscle contractile properties may be a risk factor for subsequent injury has been proposed (see references: https://pubmed.ncbi.nlm.nih.gov/34515974/, https://doi.org/10.5114/biolsport.2025.139853, https://doi.org/10.3390/ijerph19010474), there is no strong evidence from prospective studies utilizing TMG to investigate this question. Therefore, I suggest being less definitive in the introduction regarding this issue. Instead, the introduction should provide stronger evidence from existing literature to support any assumptions regarding TMG's role in injury prevention.

- Throughout the manuscript, there is a noticeable mismatch between statements and the references used to support them. For example:

- On page 3, lines 43-44: The manuscript states that Faude et al. [22] investigated the impact of neuromuscular fatigue on sprint and jump performance in elite youth soccer players, emphasizing the importance of monitoring muscle function to optimize training and prevent injuries. However, Faude et al. did not assess the impact of neuromuscular fatigue on sprint and jump performance in that publication.
- On page 3, lines 46-48: The reference to Ekstrand et al. [24] does not support the identification of hamstring injury mechanisms related to muscle imbalances; instead, it discusses injury burden and differences in injury incidence among various hamstring muscles.

To strengthen your narrative, please refer to more appropriate references that accurately support the claims made.

- When the authors refer to "during a season," do they mean the competitive season? While the discussion section mentions the competitive season (Line 168), this should be clarified earlier in the manuscript. Please specify this in the methods section and provide a rationale for why this particular period was chosen for TMG data aggregation.

- The authors' decision to compare the results of the summarized literature with the reference values provided by the software is interesting idea. However, this approach necessitates more complex models including normalization of the published values according to participants' age, playing position, and playing status, as these factors have been shown to influence TMG parameters in athletes (https://doi.org/10.3390/ijerph20020924, https://doi.org/10.3390/sports10110177, https://doi.org/10.5114/biolsport.2025.139853). Also, more insights about the reference values provided by TMG software should be presented for better interpretation of current findings. Authors mentioned in the subchapter "Statistical analyses" that "the control group mean, and standard deviation were weighted using a sample size of forward, centre, and defense soccer players creating a single group. I believe more clarification about the control group should be provided here.

- A similar article was recently published (https://doi.org/10.5114/biolsport.2025.139853) that provided summarized TMG values for all lower limb muscles and parameters in soccer players available in the literature. The findings from this published article can serve as a valuable reference point to strengthen your introduction and discussion sections, which currently lack strong arguments for both problem identification and the interpretation of the results observed.

- The limitation section warrants further improvements as there are several issues with the authors approach and statistical models used to compare reference values, that must be addressed in detail.

Minor comments:

- **Page 2, Line 22-23**: The sentence starting with "A few years ago" is out of context and does not belong here. Please consider removing or rephrasing it.
- **Page 3, Line 23**: Consider using the term "device" instead of "tool" for consistency. Ensure that the chosen term is used consistently throughout the manuscript.
- **Page 3, Line 23-26**: I suggest shortening the sentence that starts with "Nowadays, …" to improve clarity and readability.
- **Page 3, Line 27**: While soccer is the most popular sport, it is not necessarily the most demanding, either physically or psychologically. Please consider removing this statement.
- **Page 3, Line 29-30**: Reference 15 does not support the claim about the existence of muscular imbalances in soccer. There are several other instances where references do not support the corresponding statements. Please review the entire manuscript carefully to address this issue.
- **Page 3, Line 36**: Provide more details on the results of the study by Piqueras-Sanchiz et al. This will offer readers better insights into previous studies and highlight areas that warrant further investigation.
- **Page 3, Line 39**: Muscle activation is not something that can be directly assessed by TMG, although it is sometimes interpreted as such. Please be more precise here, as TMG primarily provides insights into muscle contractile (i.e., mechanical) properties.

- From the authors' perspective, how would you differentiate between muscle activation and contractile response? Please clarify this distinction.
- **Page 3, Line 40**: What is the difference between asymmetries and imbalances in this context? Be more precise, as these terms could be interpreted as interchangeable, though it seems the authors intend to convey different concepts.
