# Supplementary material for: Contractile and mechanical properties of hamstring muscles measured by the method of tensiomyography (TMG) in professional soccer players: A systematic review, meta-analysis and meta-regression
Source: PLoS One. 2025 Mar 4;20(3):e0315812. doi: 10.1371/journal.pone.0315812 (PMC11878904; doi:10.1371/journal.pone.0315812)
Supplement: S1 — (DOCX) [file pone.0315812.s002.docx]

The introduction section addresses the relevance of TMG and its utility in evaluating muscle properties. However, it lacks a structured approach and problem identification. The rationale behind focusing on hamstring muscles in soccer is not fully developed. To enhance the introduction:

- Clarify the Problem Statement: Elaborate on the importance of TMG in evaluating specific parameters critical to injury prevention, especially for soccer players prone to hamstring injuries.

Response: To clarify, it is important to note that, to date, there is only one experimental study in which an individualized training program was implemented with the specific aim of preventing injuries. This highlights the need for further research on the effectiveness of TMG in assessing key parameters that could aid in injury prevention, particularly for soccer players who are prone to hamstring injuries.

- Focus on Existing Gaps: The transition from general TMG applications to the study's objectives is abrupt. Consider discussing gaps in previous literature regarding TMG's efficacy for muscle-specific injury risk assessment.

Response: Enhanced the final segment of the introduction by incorporating additional details to better substantiate the justification of the research problem, providing a more compelling context before transitioning to the objectives.

- Discussion: Comparison with Existing Literature: The manuscript could improve by systematically comparing its findings with previous literature to contextualize the significance of specific TMG parameters. Interpretation of Tc, Td, and Dm Differences: The physiological interpretation of contraction time (Tc), muscle displacement (Dm), and delay time (Td) is crucial but underexplored. Explaining how these parameters relate to hamstring function and soccer-specific demands could enhance relevance.

Response: In the *Study Selection* and data extraction section, this required issue is explained a bit more. We added more information about Td in this section. Additionally, in the *Discussion*, we have added more information about the Tc and Dm.

Limitations: While some limitations are mentioned, the manuscript could expand on them, especially regarding the small sample sizes for certain parameters and the exclusion of Tr and Ts values. Additionally, the limited number of studies on the semitendinosus muscle and potential biases in TMG parameter selection should be acknowledged.

Response: added the suggestions

- Minor typographical errors in the results and discussion sections should be corrected.

Response: Corrected

This study presents a valuable contribution to the field of sports science and injury prevention in professional soccer. However, refinements to the methodology, expanded discussion on limitations, and improved adherence to reporting guidelines are recommended to elevate the quality and impact of the manuscript
